# Supplementary material for: Synthesis and Advanced NMR Characterization of Ordered 3D Reticular Materials with PolySilicate Nodes and Hydrophobic OrganoSilicone Linkers
Source: Molecules. 2025 Jan 8;30(2):228. doi: 10.3390/molecules30020228 (PMC11767319; doi:10.3390/molecules30020228)
Supplement: Supplementary file 1 [file molecules-30-00228-s001.zip › molecules-3407050-supplementary.pdf]

Supplementary Materials

Synthesis and Advanced NMR Characterization of Ordered 3D Reticular Materials with PolySilicate Nodes and Hydrophobic OrganoSilicone Linkers

Jelle Jamoul [1,†], Sambhu Radhakrishnan [1,2,†], Maarten Houlleberghs [1], C. Vinod Chandran [1,2], Aline Vits [1], Pasquinel Weckx [1,2,] Sam Smet [1,] Daniel Arenas Esteban [3], Sara Bals [3], Johan A. Martens [1] and Eric Breynaert [1,2,]*

[1] Centre for Surface Chemistry and Catalysis—Characterization and Application Team (COK-KAT), KU Leuven, Celestijnenlaan 200F Box 2461, 3001 Heverlee, Belgium

[2] NMRCoRe—NMR/X-Ray Platform for Convergence Research, KU Leuven, Celestijnenlaan 200F Box 2461, 3001 Heverlee, Belgium

[3] Electron Microscopy for Materials Science (EMAT), University of Antwerp, Groenenborgerlaan 171, 2020 Antwerp, Belgium

[*]Correspondence: eric.breynaert@kuleuven.be

†These authors contributed equally to this work.

List of Contents

Supplementary data

S1.1. Diffraction patterns describing PSS materials before and after calcination (Figures S1 and 2)

S1.2. NMR spectra unravelling the chemical structure of PSS materials (Figures S3 – S7 & Table S1 and S2)

S1.3. Investigation of thermal properties of PSS materials (Figures S8 – S11)

Discussions

S2.1. Assignment of resolved D-coordinated Si atoms in 1D ^{29}Si MAS NMR of PSS-4, according to their chemical functionality, based on fitting ^{1}H and ^{29}Si NMR spectra (Table S3)

S2.2. Calculations of the theoretical weight of PSS-3 and PSS-4 (Table S4)

Supplementary data

S1.1 Diffraction patterns describing PSS materials before and after calcination

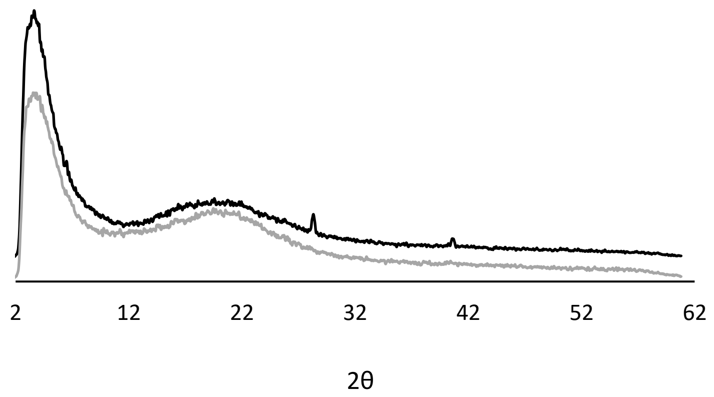

Figure S1. Diffraction patterns of PSS-3 (50% deficit silane) before (bottom) and after (top) calcination. Three reflections at 28.3, 40.5, and 50.2 2θ were detected in calcined samples but the phase causing these reflections has yet to be determined.

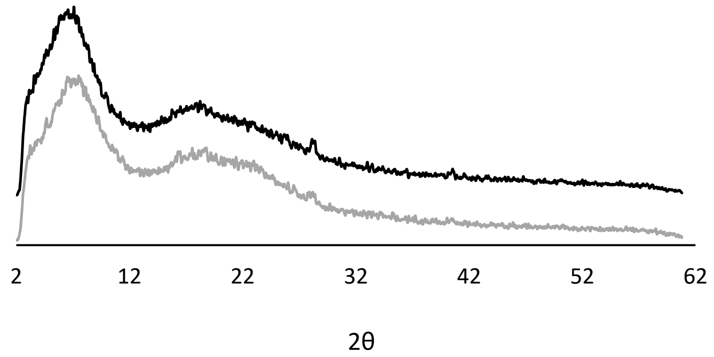

Figure S2. Diffraction patterns of PSS-4 before (bottom) and after (top) calcination. Three reflections at 28.3, 40.5, and 50.2 2θ were detected in calcined samples but the phase causing these reflections has yet to be determined.

S1.2. NMR spectra unraveling the chemical structure of PSS materials

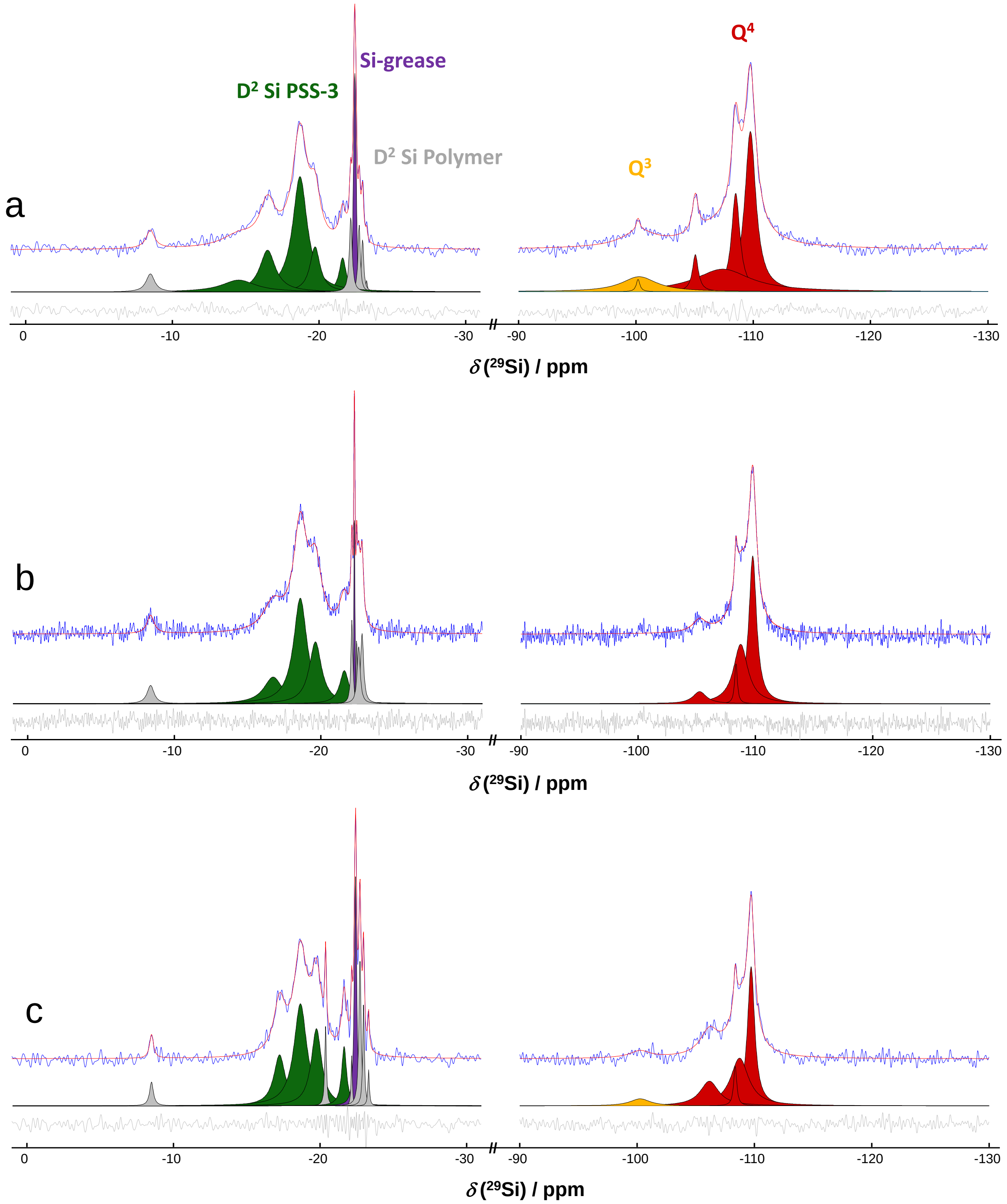

Figure S3. Decomposition of 1D ^{29}Si MAS NMR spectra of PSS-3 with a) 50% deficit, b) stoichiometric, and c) 50% excess silane concentration. Individual components are colored corresponding to the Si nature as shown in Figure 3a. Silicone grease is colored in purple in accordance to Figure 3b. The grey curves in the D Si region (0 - -30 ppm) are assigned to octyl(methyl)silicone polymer and were excluded from the calculations of the average length of the silicone linkers in Figure 3e.

Table S1. Chemical shift, signal intensity, and Si atom designation of all individual signals from Figure S3.

Chemical shift (ppm)	Integrated area	Si atom designation	Total area (Normalized per cube)
	50% Deficit		
-109,76	84377,12	Q^4	191 590,81 (7)
-108,51	39116,28	Q^4	
-107,41	57782,02	Q^4	
-105,07	10315,39	Q^4	
-100,28	25014,89	Q^3	27 161,19 (1)
-100,19	2146,3	Q^3	
-23,25	684,22	D^2	150 637,51 (5,5)
-22,97	4719,09	D^2	
-22,74	5667,68	D^2	
-22,45	15147,21	D^2	
-22,17	7901,29	D^2	
-21,62	9023,64	D^2	
-19,74	17501,42	D^2	
-18,71	64877,19	D^2	
-16,5	24988,24	D^2	
-14,53	15274,74	D^2	
-8,51	5958,58	D^1	
	Stoichiometric		
-109,76	6057,36	Q^4	12 096,66 (8)
-108,73	4571,54	Q^4	
-108,34	576,01	Q^4	
-105,24	891,75	Q^4	
-22,8	926,86	D^2	15571,51 (9,7)
-22,57	731,77	D^2	
-22,44	367,9	D^2	
-22,28	652,33	D^2	
-22,11	675,4	D^2	
-21,6	1328,6	D^2	
-19,62	3280,59	D^2	
-18,59	6106,45	D^2	
-16,74	2153,94	D^2	
-8,39	548,05	D^1	
	50% Excess		
-109,72	32129,73	Q^4	78 490,82 (7,6)
-108,74	27692,97	Q^4	
-108,37	3937,73	Q^4	
-106,17	14730,39	Q^4	
-100,27	4594,01	Q^3	4594,01 (0,4)
-23,32	1377,5	D^2	97.024,86 (9,5)
-22,97	4316,55	D^2	
-22,73	8272,95	D^2	
-22,42	13516,17	D^2	

-22,16	1566,44	D^2
-21,65	7585,11	D^2
-20,38	3337,97	D^2
-19,76	20898,37	D^2
-18,67	34350,31	D^2
-17,24	15319,66	D^2

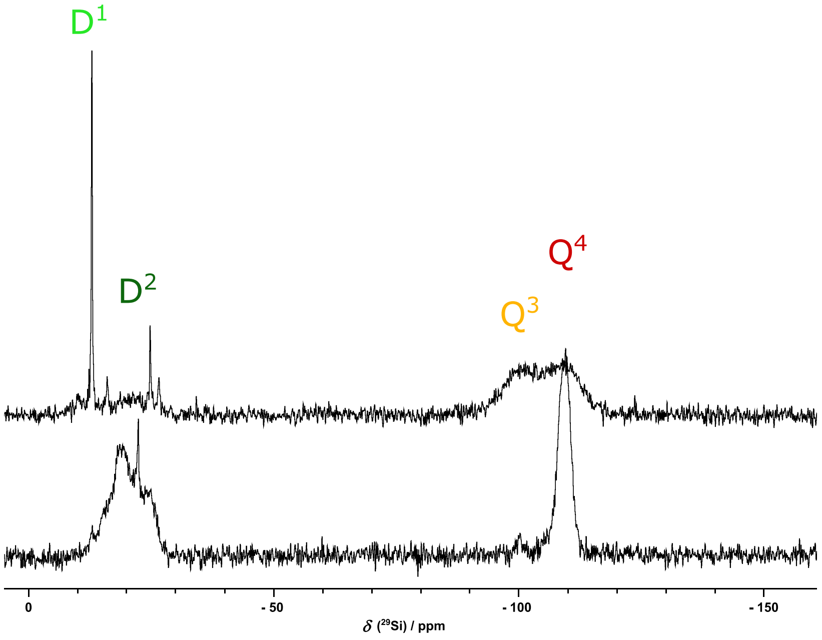

Figure S4. 1D ^{1}H decoupled ^{29}Si MAS NMR spectra of PSS materials synthesized with a 50% deficit of dicyclopentyilsilane only (top) and a mix of dimethyl- and dicyclopentylsilane with an 8 to 3 ratio respectively (bottom).

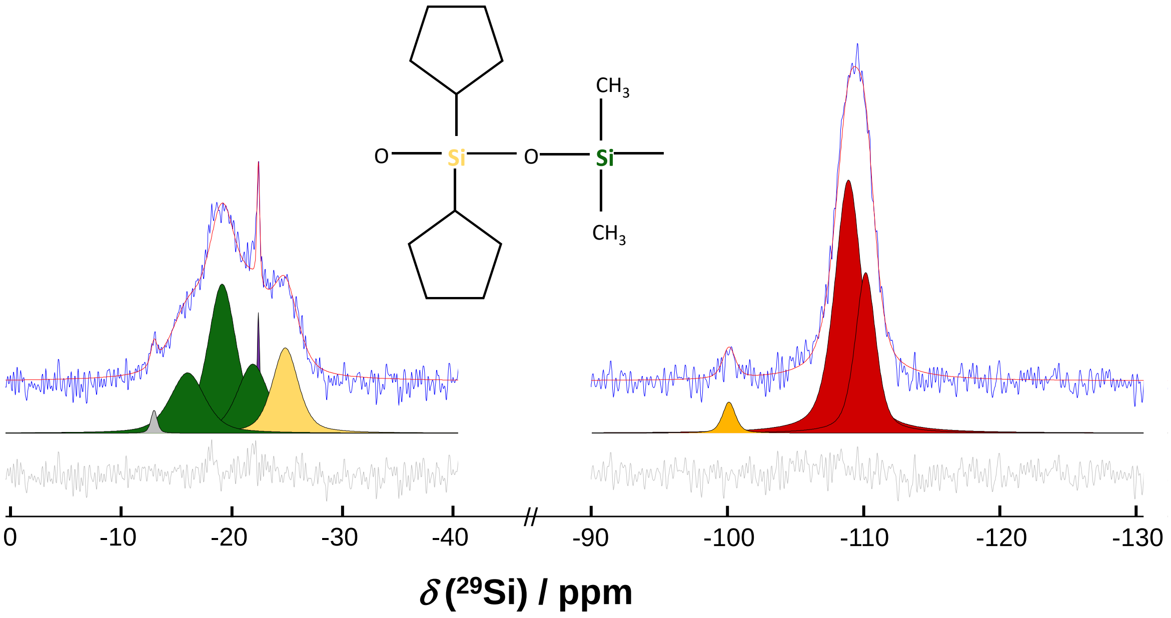

Figure S5. Decomposition of the 1D ^{1}H decoupled ^{29}Si MAS NMR spectrum of PSS-4 (blue trace), fitted spectrum (red trace), and the individual components colored corresponding to the nature of the Si atom depicted in the chemical structure model. The difference between the experimental and fitted spectrum is added (grey trace). The sharp resonance at -22.4 ppm is assigned to silicone grease contamination, used to seal the set-up airtight.

Table S2. Si speciation, chemical shift, and relative intensities per cube of all individual components fitted in the decomposition of the ^{29}Si NMR of PSS-4 in Figure S5.

Si speciation	Chemical shift (ppm)	Relative ^{29}Si intensity per cube
D^1 - $Si(Cp)_2$	-13	0.14
D^2 - $Si(CH_3)_2$	-16	2
D^2 - $Si(CH_3)_2$	-19.1	4
D^2 - $Si(CH_3)_2$	-21.9	2
D^2 - $Si(Cp)_2$	-24.8	2.1
Q^3	-100.1	0.3
Q^4	-108.9 ; -110.2	7.7

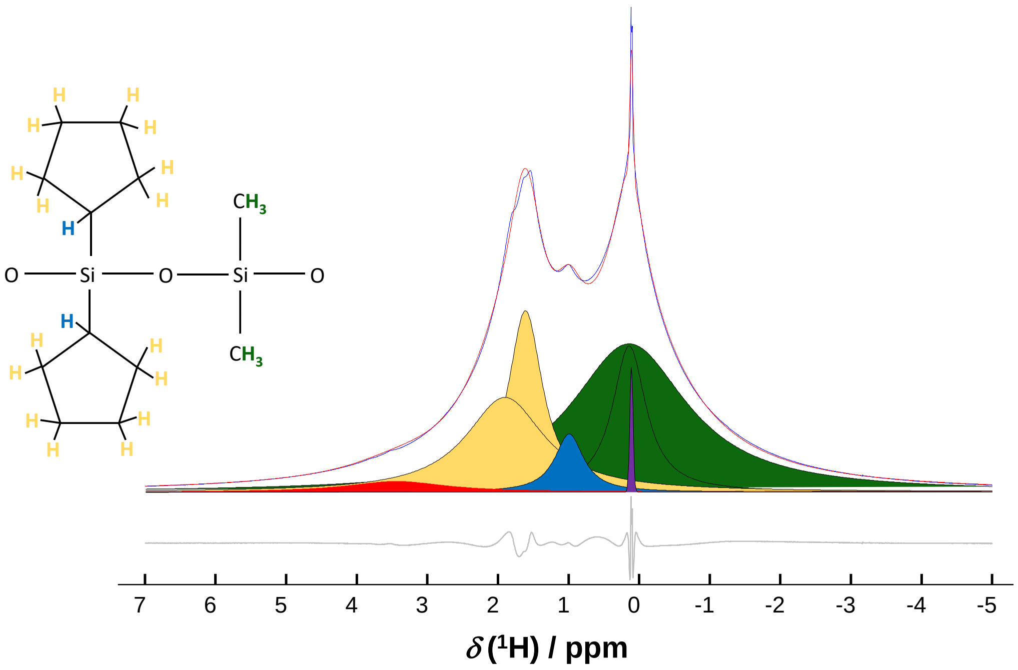

Figure S6. Decomposition of the 1D ^1H MAS NMR spectrum of PSS-4 (blue curve), fitted spectrum (red curve), and their difference (grey curve). The individual components are colored corresponding to the nature of the protons depicted in the chemical structure model. Silicone grease is colored in purple. The signal colored in red is assigned to a very small residual water fraction (only 2.7% of the total ^1H intensity of the spectrum) in chemical exchange with the silanols. The spectrum was measured at 15 kHz in a 500 MHz spectrometer.

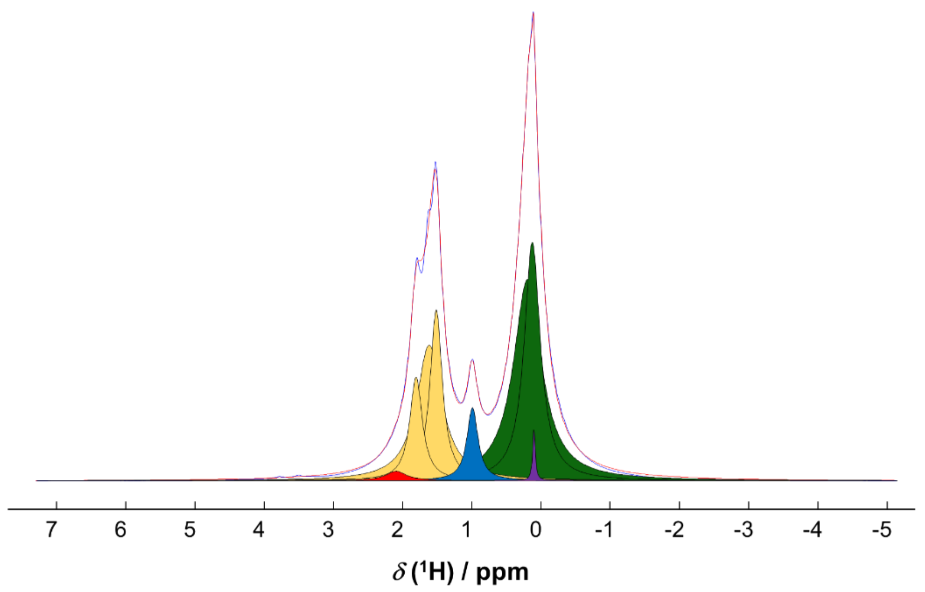

Figure S7. Decomposition of the 1D ^{1}H MAS NMR spectrum of PSS-4 (blue curve) and fitted spectrum (red curve). The individual components are colored corresponding to the nature of the protons depicted in the chemical structure model in Figure S6. Silicone grease and silanols are respectively represented by purple and red traces. The spectrum was measured at 40 kHz in a 800 MHz spectrometer.

S1.3. Investigation of thermal properties of PSS materials

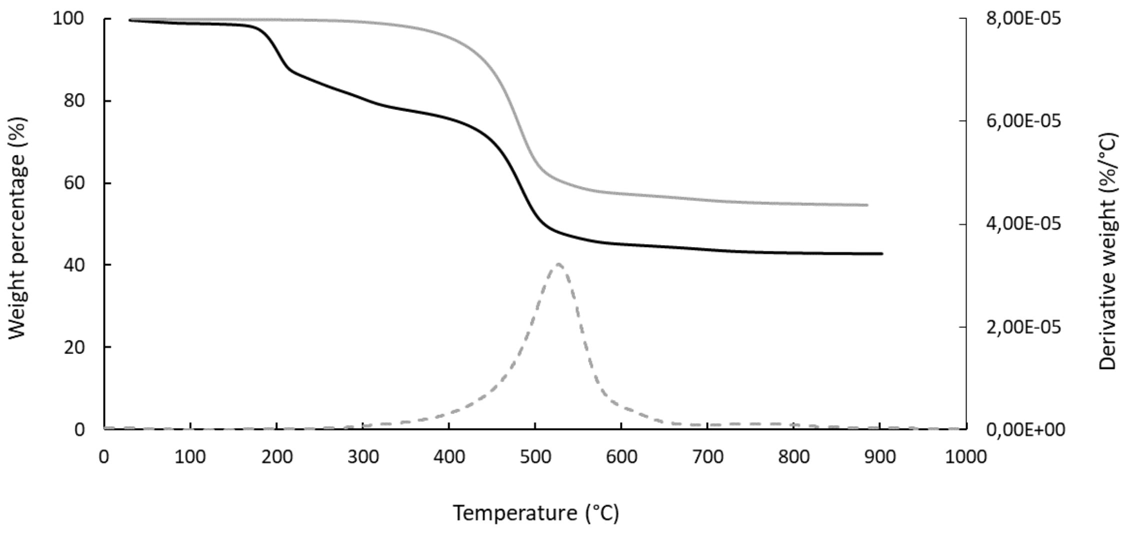

Figure S8. Thermogravimetric analysis of PSS-3 (50% deficit) in inert (N_2) atmosphere before (bottom; black trace) and after (top; grey trace) calcination. The first weight loss around 190 °C is assigned to TBA-Cl decomposition while the degradation of the organic octyl and methyl moieties initiates from 400 °C. The thermal stability of PSS-3 is maintained after calcination. The DTG curve of the calcined sample is also added and scaled for better visibility (dotted line).

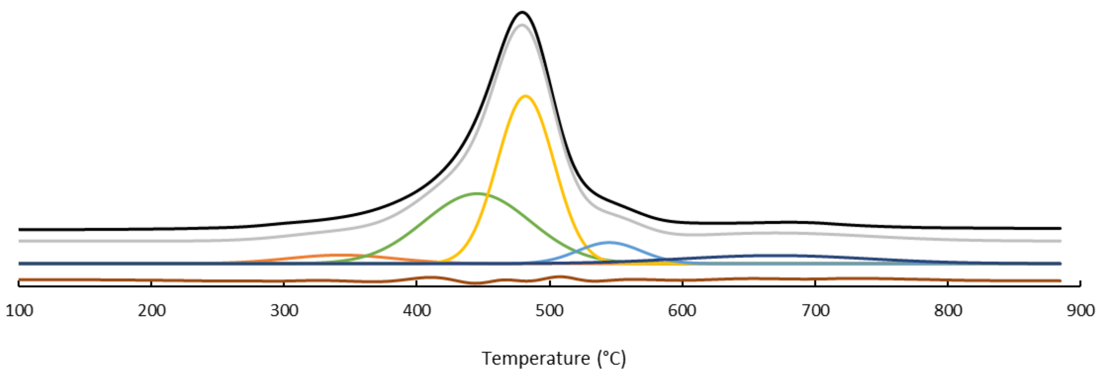

Figure S9. Decomposition of the DTG curve of a calcined PSS-3 sample. Color code: Black = DTG curve ; Grey = total plot ; brown = difference plot; orange, green, yellow, and blue = individual components.

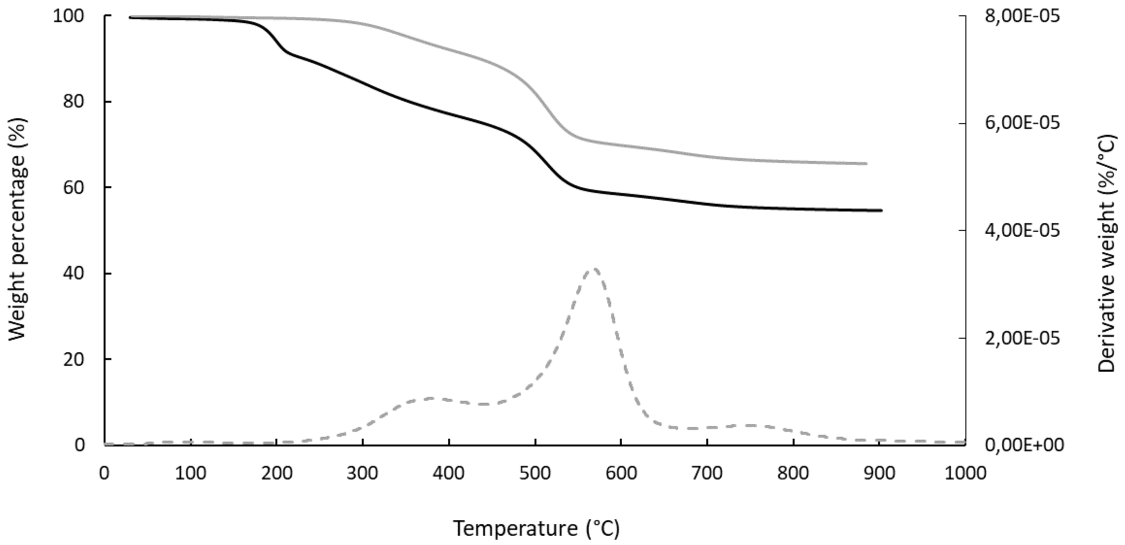

Figure S10. Thermogravimetric analysis of PSS-4 in inert (N_2) atmosphere before (bottom; black trace) and after (top; grey trace) calcination. The first weight loss around 190 °C is assigned to TBA-Cl decomposition while the degradation of the organic cyclopentyl and methyl moieties initiates from 250 °C. Thermal stability of PSS-4 is maintained after calcination. The DTG curve of the calcined sample is also added and scaled for better visibility (dotted line).

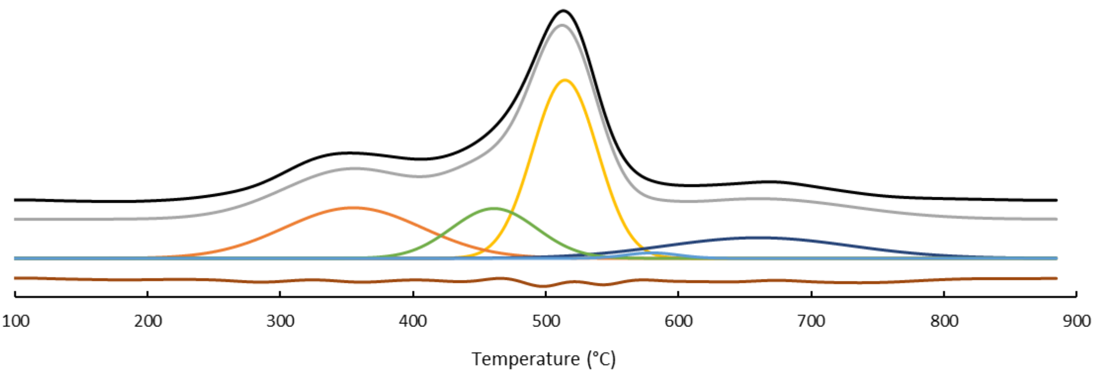

Figure S11. Decomposition of the DTG curve of a calcined PSS-4 sample. Color code: Black = DTG curve ; Grey = total plot ; brown = difference plot; orange, green, yellow, and blue = individual components.

Discussions

S2.1. Assignment of resolved D-coordinated Si atoms in 1D ^{29}Si MAS NMR of PSS-4, according to their chemical functionality, based on fitting ^{1}H and ^{29}Si NMR spectra: Decomposition of the 1D ^{29}Si MAS NMR spectrum of PSS-4 revealed multiple signals in the D^2 region of which the specification is listed in Table S1. The nature of the D^2 ^{29}Si resonance at -24.8 ppm is assigned to a $Si(Cp)_2$ moiety in the silicone linkers, based on the calculations detailed below.

1) According to the Si assignment in Table S1, the $Si(CH_3)_2/Si(Cp)_2$ ratio is 3.85.

2) According to the decomposed ^{1}H MAS NMR spectrum (Figure S6), the intensity of all resolved protons of $Si(CH_3)_2$ and $Si(Cp)_2$ are in Table S2. The ratio of the resolved cyclopentyl protons (Si-(C$\mathbf{H_2}$)$_{4\Delta}$ / Si-C**H**) is 7.95, justifying the molecular structure of cyclopentyl, and the reliability of the ^{1}H NMR fit and these calculations. Since each Si atom has a vast amount of protons, the ^{1}H intensities can be converted to Si intensities. For the Si-(C$\mathbf{H_3}$)$_2$, Si-C**H**, and Si-(C$\mathbf{H_2}$)$_{4\Delta}$, the conversion factor is 6, 2, and 16 respectively, which is derived from their molecular structure. From the calculated Si intensities, the $Si(CH_3)_2/Si(Cp)_2$ ratio is 4.1. From the decomposition of the ^{1}H NMR spectrum measured at 40 kHz in a 800 MHz spectrometer (Figure S7), the $Si(CH_3)_2/Si(Cp)_2$ ratio is 3.87.

   Both $Si(CH_3)_2/Si(Cp)_2$ ratio's (from 1) & 2)) are in good agreement with each other, justifying the assignment of $Si(Cp)_2$ silicone moieties resonating at -24.8 ppm.

Table S3. Details of individual proton components according to Figure S6. The proton intensities can be converted to silicon intensities based on the numbers of protons per Si derived from the molecular structure of the methyl and cyclopentyl functional groups.

Proton speciation	Proton intensity	Si intensity
Cyclopentyl		
Si-C**H**	60669	30334.5
Si-(C$\mathbf{H_2}$)$_{4\Delta}$	482621	30164
Methyl		
Si-(C$\mathbf{H_3}$)$_2$	748133	124689

S2.2. Calculations of the theoretical weight of PSS-3 and PSS-4: The theoretical weight percentage (wt%) of the organic functional groups of PSS-3 and PSS-4 can be calculated from the molecular composition (Figure 3e). The PSS-4 sample was selected for a detailed calculation. The molecular weight of the individual components, *i.e.,* silicate cube and silicone linkers, is listed in Table S3. The same approach can be used for PSS-3.

1. The organic weight fraction:

   The total organic weight of PSS-4 = (2* (30+138)) + (4*45) g/mol = 516 g/mol.

   The 1st factor of the summation is the organic content of the 4 dimeric linkers, consisting of Me_2 and Cp_2 mixed silicone linker of -O-$Si(CH_3)_2$-O-$Si(C_5H_9)_2$-O-. Dimethyl and dicyclopentyl functionalities have a total molecular weight of 30 and 138 g/mol, respectively. The average contribution of each dimethyl and dicyclopentyl groups per cube is half of the numbers of dimeric linkers, *i.e.,* 2.

   The 2nd factor of the summation is the organic content of the trimeric silicone linkers with only dimethyl groups attached to the Si centers. In total, there are 4 trimeric linkers per cube of which each trimeric linker contributes 1.5 dimethyl groups per cube with a total molecular weight of 45 g/mol.

2. Inorganic weight fraction:

   The total inorganic weight of PSS-4 = 544.64 + 4* (28.08 + 8) + 4* (42.12 + 16) g/mol = 921.44 g/mol.

   It is assumed that the remaining inorganic fraction of PSS-4 is silica, without any organic species. The inorganic fraction is constituted of the silicate cube (Si_8O_{20}, first factor) and the Si-O backbone of the silicone linkers (2nd and 3th factor).

   Considering one functionalized silicate cube of PSS-4, the total weight is equal to the sum of the organic fraction (516 g/mol) and the inorganic fraction (921.44 g/mol), being 1437.44 g/mol. The organic content is 35.9 wt% ($= \frac{516\text{ g/mol}}{1437.44\text{ g/mol}}$ * 100) and the inorganic content is 64.1 wt% ($= \frac{921.44\text{ g/mol}}{1437.44\text{ g/mol}}$ * 100).

Table S4. Molecular weight of all individual components of PSS-3 and PSS-4, based on the composition of Figure 3e. The composition of the silicone linker is on average per silicate cube. These numbers were used to calculate the weight percentage of the organic functionalities of PSS-3 and PSS-4 to justify the measured values obtained via thermogravimetric analysis.

Individual components	Molecular composition		Molecular weight	
	PSS-3 (50% deficit)	PSS-4	PSS-3 (50% deficit)	PSS-4
Silicate cube	Si_8O_{20}	Si_8O_{20}	544.64	544.64
Silicone linkers				
Monomeric	$(Si(C_8H_{17})(CH_3))_{0.5}$	/	78.04	/
Dimeric	$Si(C_8H_{17})(CH_3)O_{0.5}$	$Si(CH_3)_2O_{0.5}$ & $Si(C_5H_9)_2O_{0.5}$	164.08	240.16
Trimeric	/	$(Si(CH_3)_2)_{1.5}O$	/	103.12
